# Type III uveal effusion syndrome suspected to be related to pachychoroid spectrum disease: A case report

**DOI:** 10.1097/MD.0000000000021441

**Published:** 2020-07-31

**Authors:** Yuya Terubayashi, Seita Morishita, Ryohsuke Kohmoto, Masashi Mimura, Masanori Fukumoto, Takaki Sato, Takatoshi Kobayashi, Teruyo Kida, Tsunehiko Ikeda

**Affiliations:** Department of Ophthalmology, Osaka Medical College, Takatsuki-City, Osaka, Japan.

**Keywords:** optical coherence tomography, pachychoroid spectrum disease, retinal detachment, sclerotomy, uveal effusion syndrome, vitrectomy

## Abstract

**Introduction:**

We report a case of type III uveal effusion syndrome (UES) suspected to be related to pachychoroid spectrum disease.

**Patient concerns:**

A 42-year-old man became aware of visual field constriction and deterioration of visual acuity in his right eye.

**Diagnosis:**

Upon examination, a bullous non-rhegmatogenous retinal detachment was observed in the inferior 2 quadrants of the right eye fundus, and the subretinal fluid moved with postural changes. The axial length in that eye was 22.36 mm, thus indicating no nanophthalmia. Preoperative indocyanine green angiography revealed dilated choroidal vessels in the posterior pole of the right eye and mild leakage in the late phase. Optical coherence tomography examination revealed choroidal thickening in both eyes.

**Interventions:**

For treatment, we first performed sclerotomy, and the intraoperative findings showed no thickening of the sclera. Following surgery, reattachment of the retina was not achieved.

**Outcomes:**

Thus, we next performed vitrectomy, which led to successful reattachment of the retina.

**Lessons:**

In this case, we theorize that pachychoroid spectrum disease might have been involved in the pathogenesis of type III UES.

## 1 Introduction

Uveal effusion syndrome (UES) is a non-rhegmatogenous retinal detachment (RD) in which the subretinal fluid reportedly moves with postural changes.^[1,2]^ Possible causes include accumulation of exudates from choroidal vessels due to the compression of vortex veins by the thickened sclera, and impaired outflow of intraocular fluid due to decreased scleral permeability. However, the pathogenesis of UES without nanophthalmia or scleral thickening has yet to be fully elucidated. Here we report a case of UES without nanophthalmia in which sclerotomy failed and retinal reattachment was achieved via vitrectomy. Optical coherence tomography (OCT) findings in this case suggested a possible association with pachychoroid spectrum disease.

## 2 Case report

**Figure 1:**
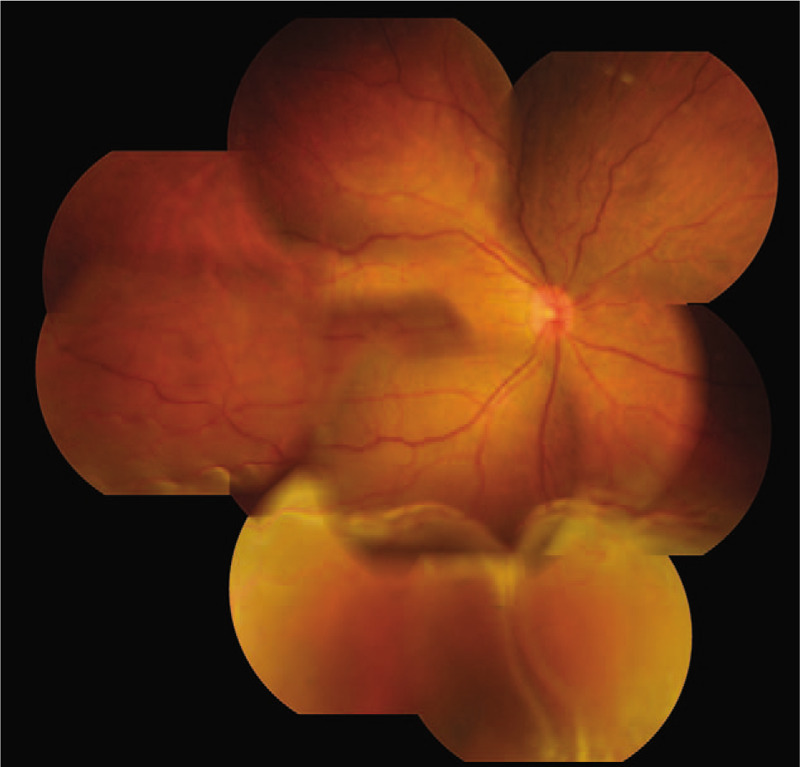
Fundus photograph of the patient's right eye obtained at the initial examination. A bullous non-rhegmatogenous retinal detachment (RD) was observed in the inferior 2 quadrants of the right fundus, and the subretinal fluid moved with postural changes.

**Figure 2:**
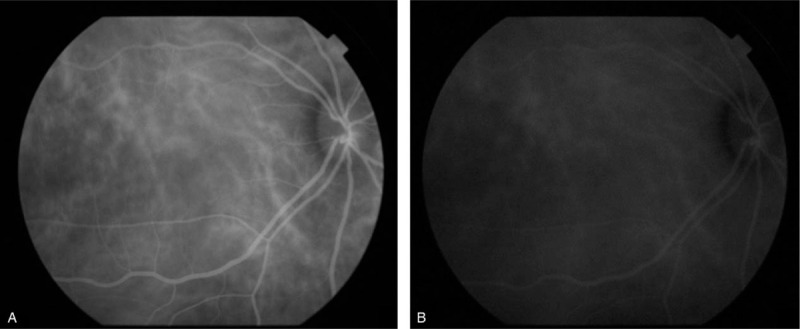
Indocyanine green angiography of the right eye performed at the initial examination. Dilated choroidal vessels in the posterior pole (A) and leakage in the late phase (B) were observed in the right eye.

**Figure 3:**
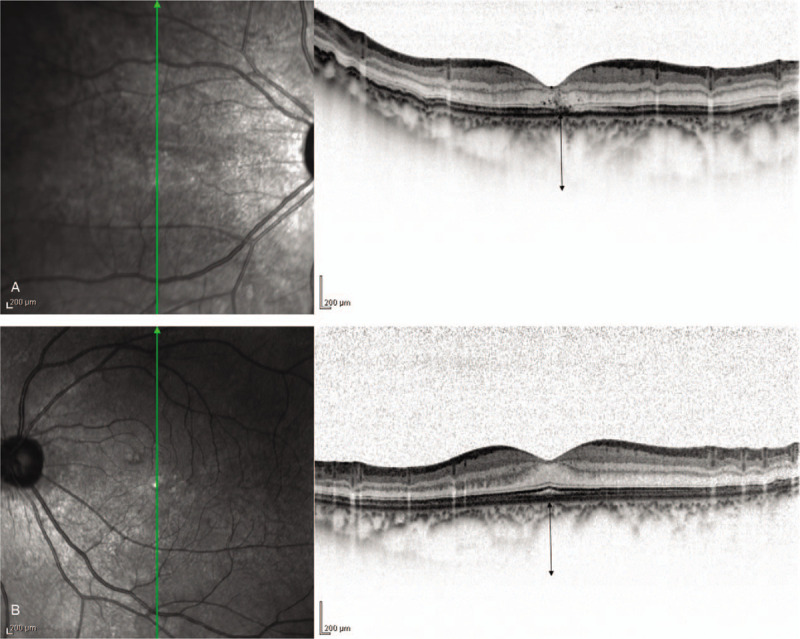
Optical coherence tomography (OCT) image obtained post vitreous surgery (right eye: A, left eye: B). Choroidal thickening were observed in both eyes. The outer layer showed highly dilated choroidal vessels, and the inner layer showed compressed choroidal capillaries. The choroidal thickness in the posterior pole of the right eye and left eye was approximately 458 and 452 μm, respectively.

A 45-year-old man became aware of visual field constriction and deterioration of visual acuity (VA) in his right eye around mid-November 2015, and presented at our department on December 7, 2015, nearly 1 month later. Upon initial examination of his right and left eye, the corrected VA was 0.15 and 1.5, respectively, and the intraocular pressure was 4 and 12 mm Hg, respectively. A bullous non-rhegmatogenous RD was observed in the inferior 2 quadrants of the right-eye fundus, and the subretinal fluid moved with postural changes; that is, was “mobile” (Fig. 1). The axial length in his right and left eye was 22.36 and 26.19 mm, respectively. Examination by fluorescein angiography (FA) revealed no abnormalities, while that by indocyanine green angiography (IA) revealed dilated choroidal vessels in the posterior pole of the patient's right eye and mild leakage in the late phase (Fig. 2A and B). OCT findings revealed bilateral choroidal thickening. The outer layer showed highly dilated choroidal vessels, and the inner layer showed compressed choroidal capillaries. The choroidal thickness in the posterior pole of the patient's right and left eye was approximately 458 and 452 μm, respectively (Fig. 3A and B). The superior choroid was found to be thicker than the inferior choroid. The color of the retinal pigment epithelium in the posterior pole of the patient's right eye was slightly uneven, thus indicating mild “leopard spot” retinopathy. No particular systemic disease was present, and the patient's past medical and family history was unremarkable.

**Figure 4:**
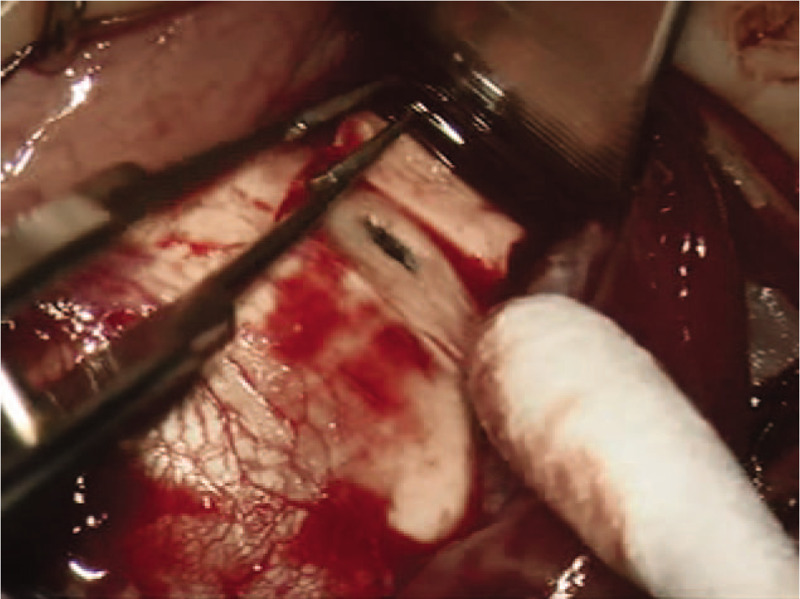
Intraoperative findings of the patient's right eye. The scleral thickness was within the normal range.

**Figure 5:**
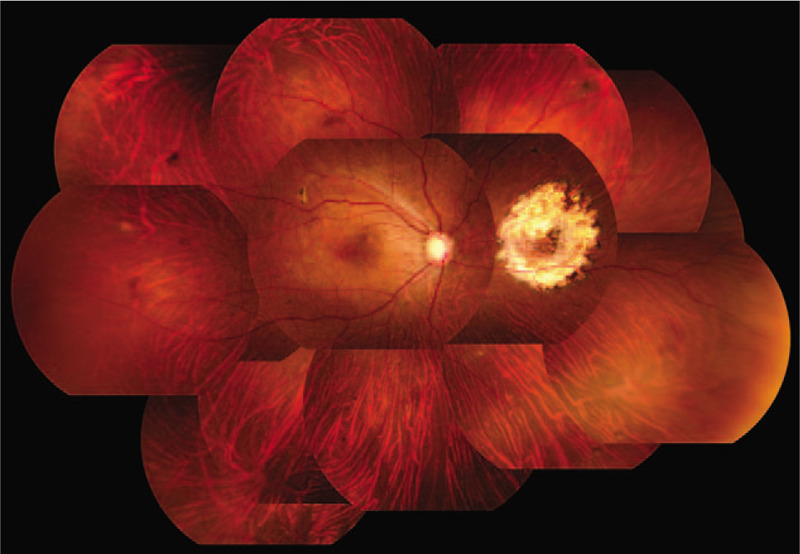
Fundus photograph of the patient's right eye obtained after vitreous surgery. The non-rhegmatogenous RD was resolved, and the corrected visual acuity improved from 0.15 to 0.5. RD = retinal detachment.

On December 15, 2015, sclerotomy was performed in 4 quadrants of the right eye. Intraoperative findings suggested that the scleral thickness was within the normal range (Fig. 4). Although the subretinal fluid decreased post surgery, it later gradually increased. Thus, vitrectomy, phacoemulsification, and silicone oil tamponade were performed on the patient's right eye on April 26, 2016. Subsequently, the silicone oil was removed and the patient underwent secondary intraocular lens implantation on July 22, 2016. The intraocular pressure improved to 12 mm Hg and corrected VA also improved to 0.5 without recurrence after surgery (Fig. 5).

This case study was approved by the Ethics Committee of Osaka Medical College, Takatsuki City, Japan, and was performed in accordance with the tenets set forth in the Declaration of Helsinki.

## 3 Discussion

In a previous study by Uyama et al,^[3]^ the authors classified UES into 3 subgroups (i.e., type I, type II, and type III) via the presence or absence of nanophthalmia and scleral thickening. In that study, type I was defined as having both nanophthalmia and scleral thickening, type II was defined as having scleral thickening without nanophthalmia, and type III was defined as having neither nanophthalmia nor scleral thickening. Sclerotomy is usually effective for the treatment of type I and type II UES, yet is often ineffective for the treatment of type III UES. Based on the mechanism of the onset of UES, type I and type II patients are considered to have choroidal hypoperfusion due to scleral thickening, resulting in retinal pigment epithelial disorder and subsequent exudative RD. Sclerotomy is considered to ameliorate the impaired tissue fluid outflow and choroidal hypoperfusion, resulting in a reduction of the subretinal fluid. However, the exact mechanism of the onset of type III UES has yet to be elucidated. Uyama et al^[3]^ reported that histological abnormalities such as the disorganization of collagen fiber bundles and deposits of glycosaminoglycan were uncommon in type III UES, and that autopsy results revealed degeneration of the retinal pigment epithelial. Those findings suggest that type III UES may be a completely different clinical entity from type I and type II UES.

In a recently study by Cheung et al,^[4]^ the authors made an attempt to redefine the disease concept of age-related macular degeneration, polypoid choroidal vasculopathy, and central serous chorioretinopathy, and stated a hypothesis that these diseases may belong to the same spectrum based on their common characteristic of choroid thickening (pachychoroid) and should thus be termed pachychoroid spectrum disease.

The OCT findings in our patient showed highly dilated choroidal vessels and compressed choroidal capillaries in both eyes, particularly in the right eye with UES. In the right and left eye, the subfoveal choroidal thickness was 550 and 500 μm, respectively. FA revealed no notable abnormal findings, while IA showed dilatation of the posterior choroidal vessels and mild leakage in the late phase, which was also consistent with the characteristics of pachychoroid spectrum disease.

To the best of our knowledge, there have been no previous reports that specifically described the association between type III UES and pachychoroid spectrum disease. However, there have been reports on cases in which patients with thickened choroid developed UES. Harada et al^[5]^ reported UES in a 41-year-old man with a marked choroidal thickening of 787 μm on spectral domain OCT and low reflection of the outer choroidal layer, and pointed out that an increase in choroidal thickness may be related to the onset of UES. Jabbarpoor Bonyadi et al^[6]^ reported a case of idiopathic UES with bullous non-rhegmatogenous RD associated with leopard spot retinopathy. Since the OCT findings in that study revealed subfoveal choroidal thickening, they speculated that it may be categorized as pachychoroid spectrum disease.

It remains an open question as to whether or not a thick choroid alone without scleral thickening causes bullous non-rhegmatogenous RD such as UES. However, Mikhail et al^[7]^ reported that sclerotomy was a useful treatment for reattachment in a patient with pachychoroid spectrum disease accompanied by a retinal pigment epithelial tear in the superior sclera and bullous non-rhegmatogenous RD in the inferior sclera. Colakoglu and Cosar^[8]^ reported the case of a 42-year-old man with atypical central serous chorioretinopathy associated with extensive serous RD, and suggested an association with pachychoroid spectrum disease.

Since the patient in this current study presented findings of suspected leopard spot retinopathy, and although not typical, it is necessary to examine the relationship between leopard spot retinopathy and pachychoroid spectrum disease. Based on the above results, we cannot rule out the possibility that type III UES, unlike type I and type II UES, is a disease that falls under the category of pachychoroid spectrum disease. Therefore, our results show that choroidal thickness should be measured in patients suspected of having type III UES via the OCT findings, and that the relationship with pachychoroid spectrum disease should be examined in a larger number of patients.

## Acknowledgments

The authors wish to thank John Bush for editing the manuscript.

## Author contributions

**Conceptualization:** Yuya Terubayashi, Seita Morishita, Tsunehiko Ikeda.

**Data curation:** Yuya Terubayashi, Seita Morishita, Masashi Mimura, Tsunehiko Ikeda.

**Formal analysis:** Yuya Terubayashi, Seita Morishita, Tsunehiko Ikeda.

**Investigation:** Yuya Terubayashi, Seita Morishita, Tsunehiko Ikeda.

**Methodology:** Yuya Terubayashi, Seita Morishita, Masanori Fukumoto, Tsunehiko Ikeda.

**Resources:** Yuya Terubayashi.

**Supervision:** Seita Morishita, Ryohsuke Kohmoto, Masanori Fukumoto, Takaki Sato, Takatoshi Kobayashi, Teruyo Kida, Tsunehiko Ikeda.

**Validation:** Tsunehiko Ikeda.

**Visualization:** Yuya Terubayashi.

**Writing – original draft:** Yuya Terubayashi, Ryohsuke Kohmoto, Masashi Mimura.

**Writing – review & editing:** Masanori Fukumoto, Takaki Sato, Takatoshi Kobayashi, Teruyo Kida, Tsunehiko Ikeda.
